# Supplementary material for: Fecal Microbial Diversity of Coyotes and Wild Hogs in Texas Panhandle, USA
Source: Microorganisms. 2023 Apr 27;11(5):1137. doi: 10.3390/microorganisms11051137 (PMC10221396; doi:10.3390/microorganisms11051137)
Supplement: Supplementary file 1 [file microorganisms-11-01137-s001.zip › microorganisms-2331569-supplementary.pdf]

Supplementary Material

Fecal Microbial Diversity of Coyotes and Wild Hogs in Texas Panhandle, USA

Babafela Awosile, Chiquito Crasto, Md. Kaisar Rahman, Ian Daniel, SaraBeth Boggan, Ashley Steuer and Jason Fritzler

Supplementary Figures

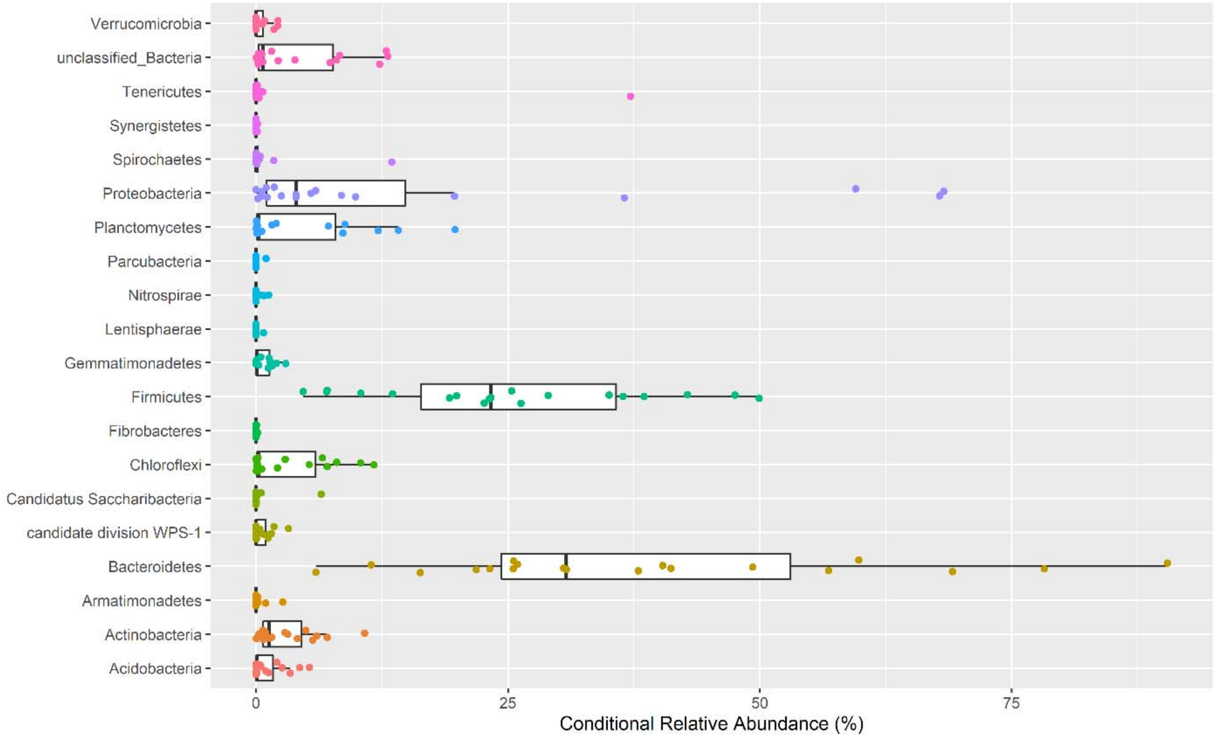

Figure S1. Boxplot distribution of relative abundance (%) of each bacteria phylum observed between the 19 Coyote samples. The plot showed *Bacteroidetes, Firmicutes,* and *Proteobacteria* as the predominant phyla in fecal microbiome of coyotes in Texas panhandle.

(**a**)

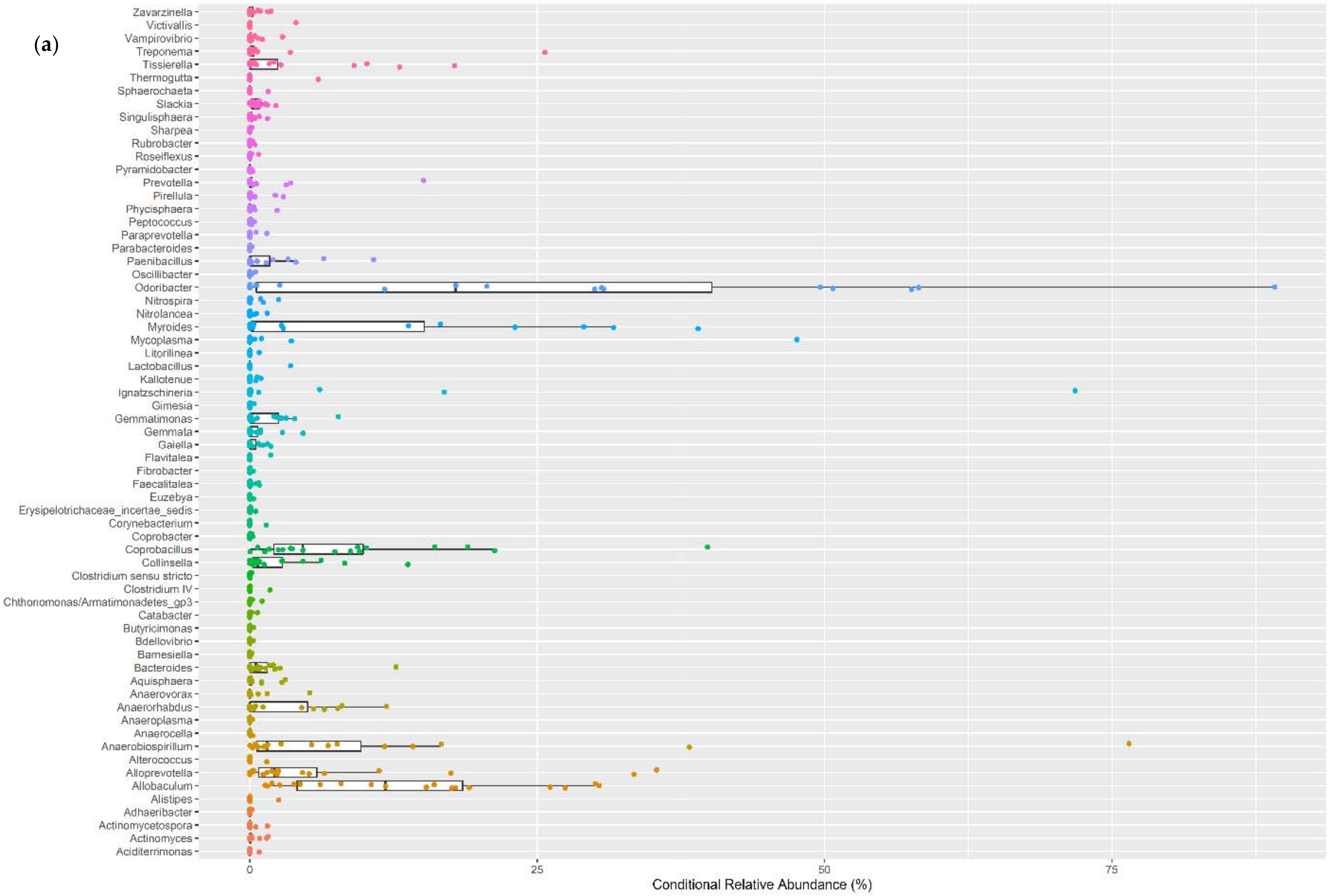
Zavarzinella
Victivallis
Vampirovibrio
Treponema
Tissierella
Thermogutta
Sphaerochaeta
Slackia
Singulisphaera
Sharpea
Rubrobacter
Roseiflexus
Pyramidobacter
Prevotella
Pirellula
Phycisphaera
Peptococcus
Paraprevotella
Parabacteroides
Paenibacillus
Oscillibacter
Odoribacter
Nitrospira
Nitrolancea
Myroides
Mycoplasma
Litorilinea
Lactobacillus
Kallotenue
Ignatzschineria
Gimesia
Gemmatimonas
Gemmata
Gaiella
Flavitalea
Fibrobacter
Faecalitalea
Euzebya
Erysipelotrichaceae_incertae_sedis
Corynebacterium
Coprobacter
Coprobacillus
Collinsella
Clostridium sensu stricto
Clostridium IV
Chthonomonas/Armatimonadetes_gp3
Catabacter
Butyricimonas
Bdellovibrio
Barnesiella
Bacteroides
Aquisphaera
Anaerovorax
Anaerorhabdus
Anaeroplasma
Anaerocella
Anaerobiospirillum
Alterococcus
Alloprevotella
Allobaculum
Alistipes
Adhaeribacter
Actinomycetospora
Actinomyces
Aciditerrimonas
0
25
50
75
Conditional Relative Abundance (%)

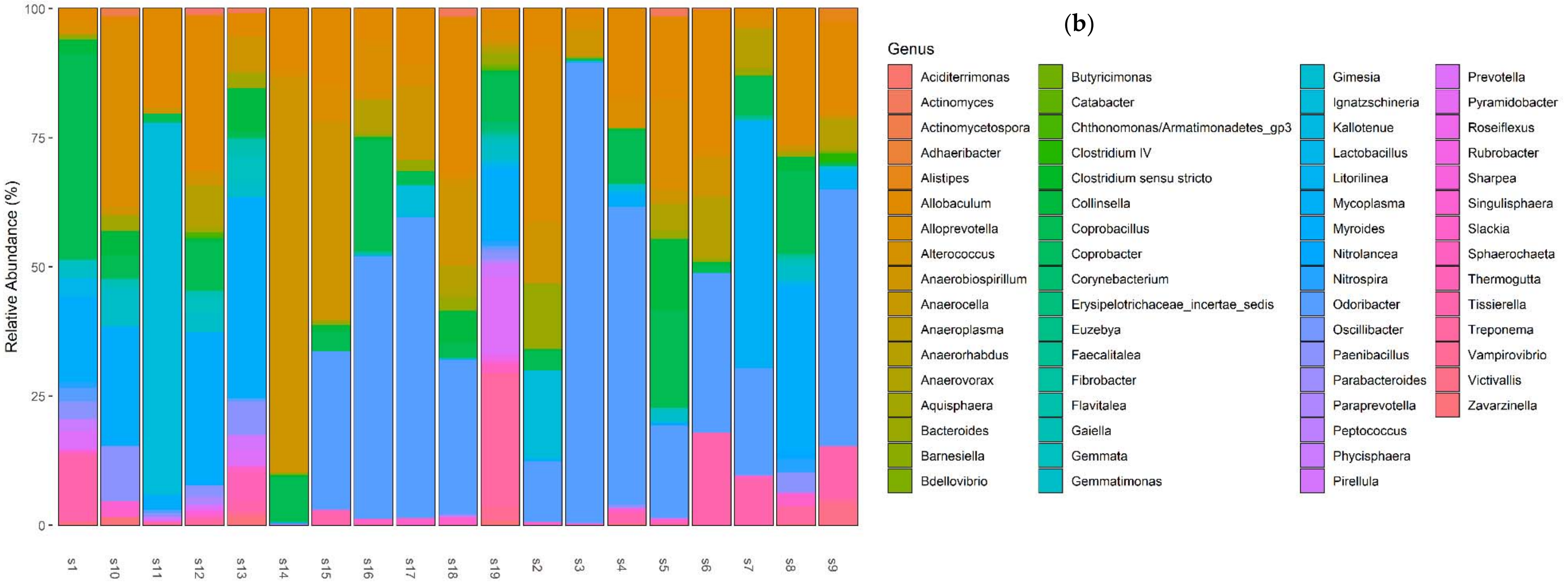

Figure S2. (**a**) Boxplot distribution of genus level relative abundance (%) observed in each 19 Coyote samples. The plot showed *Odoribacter* spp., *Allobaculum* spp., *Coprobacillus* spp., and *Alloprevotella* spp. as the dominant genera in fecal microbiome of coyotes in Texas panhandle. Data on unclassified genera were included in this plot. (**b**): Boxplot distribution of genus level relative abundance (%) observed in each 19 Coyote samples. The plot showed *Odoribacter* spp., *Allobaculum* spp., *Coprobacillus* spp., and *Alloprevotella* spp. as the dominant genera in fecal microbiome of coyotes in Texas panhandle. Data on unclassified genera were included in this plot.

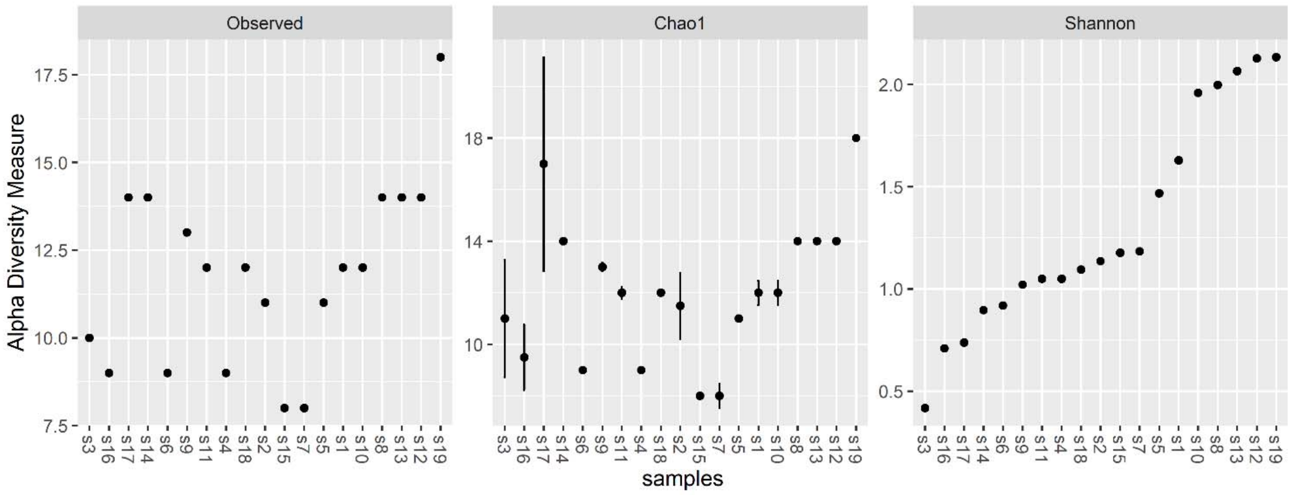

Figure S3. Alpha Diversity Index using Chao's and Shannon Indices as a measure of microbial richness and evenness within each of 19 coyote samples. Higher alpha diversity measure represents sample(s) with greater microbial specie richness and/or evenness.

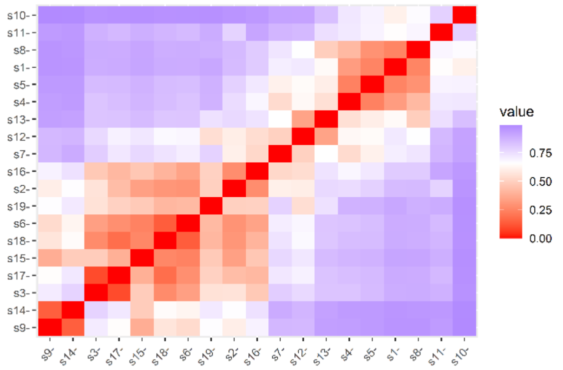

(**A**)

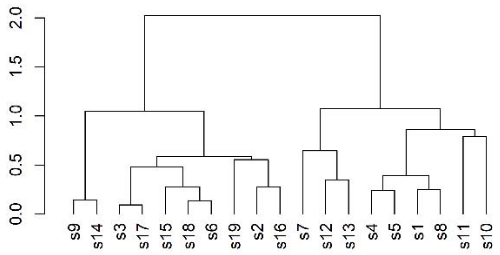

(**B**)

Figure S4. (**A**) Bray-Curtis Dissimilarity matrix and (**B**) hierarchical clustering dendrogram as measures of beta-diversity index of Coyote fecal microbiota. **(A)** Value close to 1 showed two sample are dissimilar in microbial diversity and value close to 0 show correlation and similarity between two samples. **(B)** the higher the Ward's minimum variance value between two samples, the farther the clustering compared to another one or more samples.

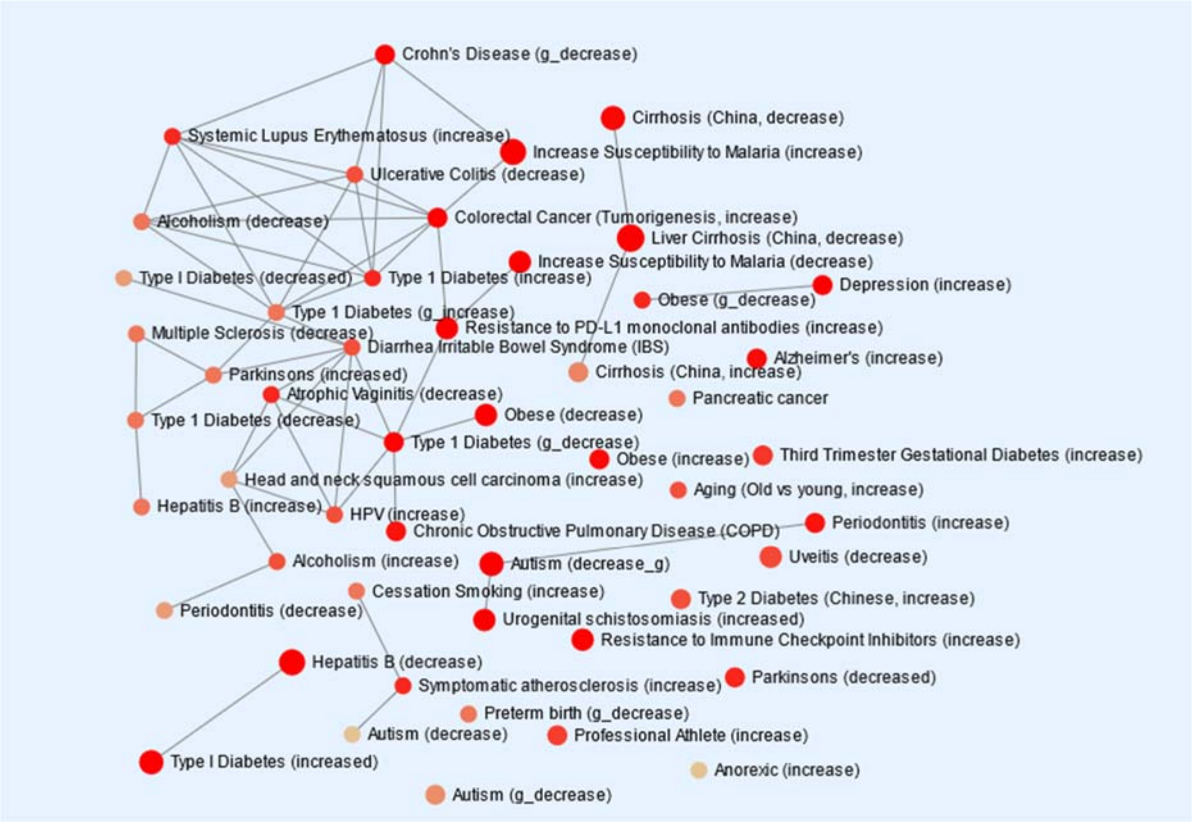

Figure S5. Predictive functional profile of coyote microbiota in relation to certain human related diseases. Each node represents a taxon set with its color based on its p-value and its size based on the number of hits in the query. Two taxon sets are connected by an edge if the number of shared taxon hits is over 20% of the total number of their combined taxa (Chong et al., 2020).

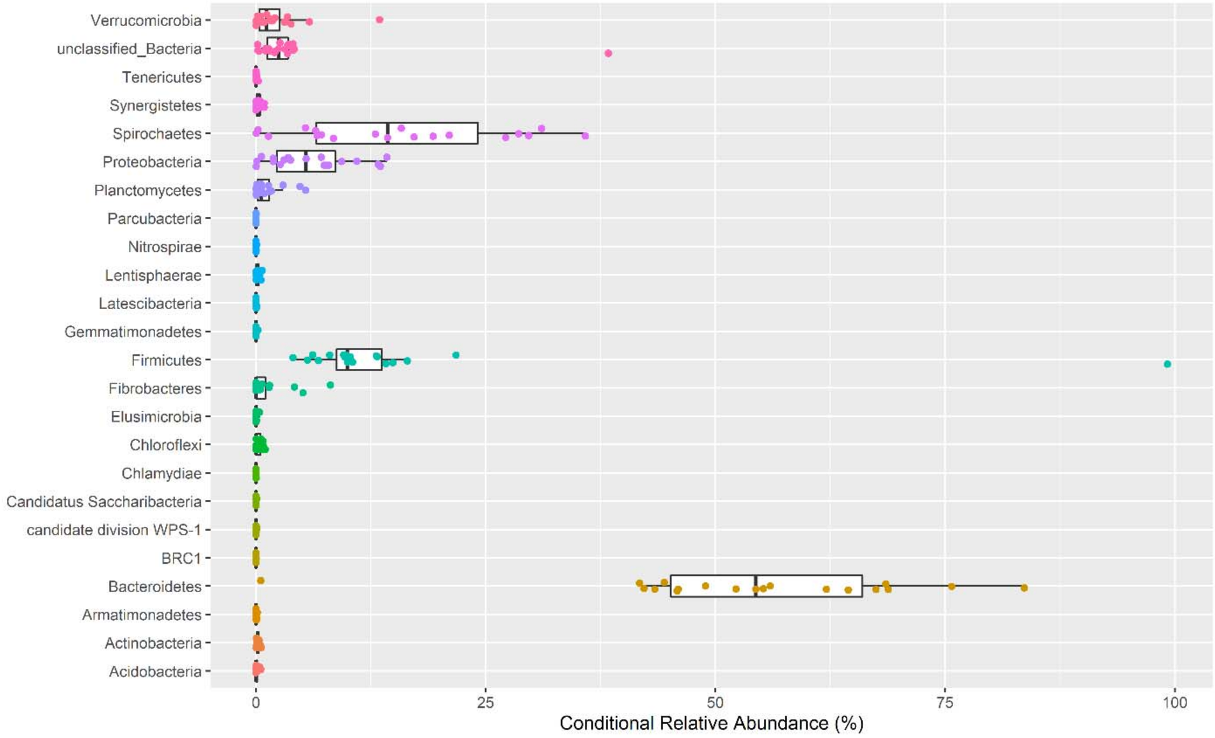

Figure S6. Boxplot distribution of relative abundance (%) of each bacteria phylum observed between the 19 wild hog samples. The plot showed *Bacteroidetes, Firmicutes,* and *Spirochaetes* as the predominant phyla in fecal microbiome of wild hogs in Texas panhandle.

(**a**)

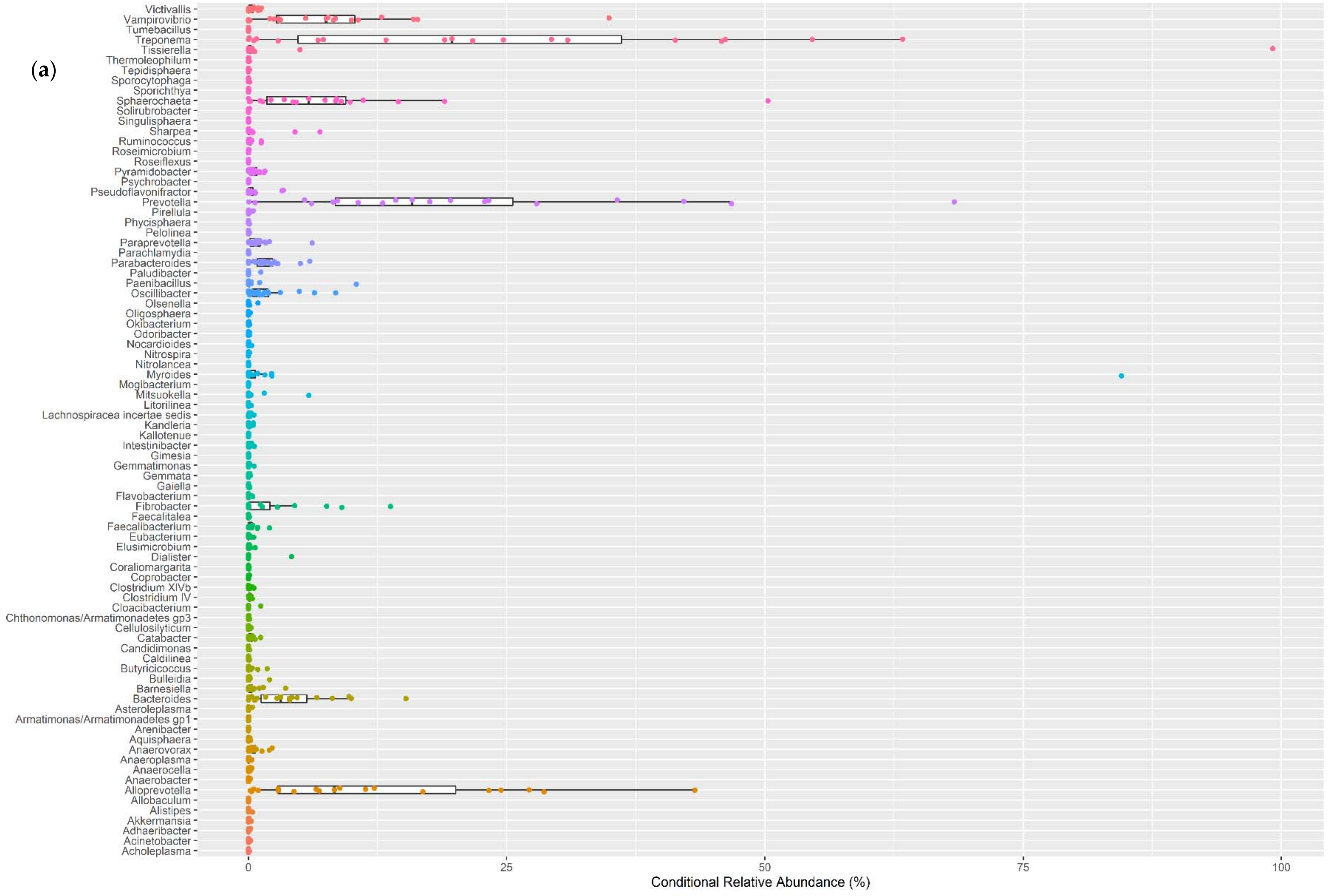
Victivallis
Vampirovibrio
Tumebacillus
Treponema
Tissierella
Thermoleophilum
Tepidisphaera
Sporocytophaga
Sporichthya
Sphaerochaeta
Solirubrobacter
Singulisphaera
Sharpea
Ruminococcus
Roseimicrobium
Roseiflexus
Pyramidobacter
Psychrobacter
Pseudoflavonifractor
Prevotella
Pirellula
Phycisphaera
Pelolinea
Paraprevotella
Parachlamydia
Parabacteroides
Paludibacter
Paenibacillus
Oscillibacter
Olsenella
Oligosphaera
Okibacterium
Odoribacter
Nocardioides
Nitrospira
Nitrolancea
Myroides
Mogibacterium
Mitsuokella
Litorilinea
Lachnospiracea incertae sedis
Kandleria
Kallotenue
Intestinibacter
Gimesia
Gemmatimonas
Gemmata
Gaiella
Flavobacterium
Fibrobacter
Faecalitalea
Faecalibacterium
Eubacterium
Elusimicrobium
Dialister
Coraliomargarita
Coprobacter
Clostridium XIVb
Clostridium IV
Cloacibacterium
Chthonomonas/Armatimonadetes gp3
Cellulosilyticum
Catabacter
Candidimonas
Caldilinea
Butyricicoccus
Bulleidia
Barnesiella
Bacteroides
Asteroleplasma
Armatimonas/Armatimonadetes gp1
Arenibacter
Aquisphaera
Anaerovorax
Anaeroplasma
Anaerocella
Anaerobacter
Alloprevotella
Allobaculum
Alistipes
Akkermansia
Adhaeribacter
Acinetobacter
Acholeplasma
0
25
50
75
100
Conditional Relative Abundance (%)

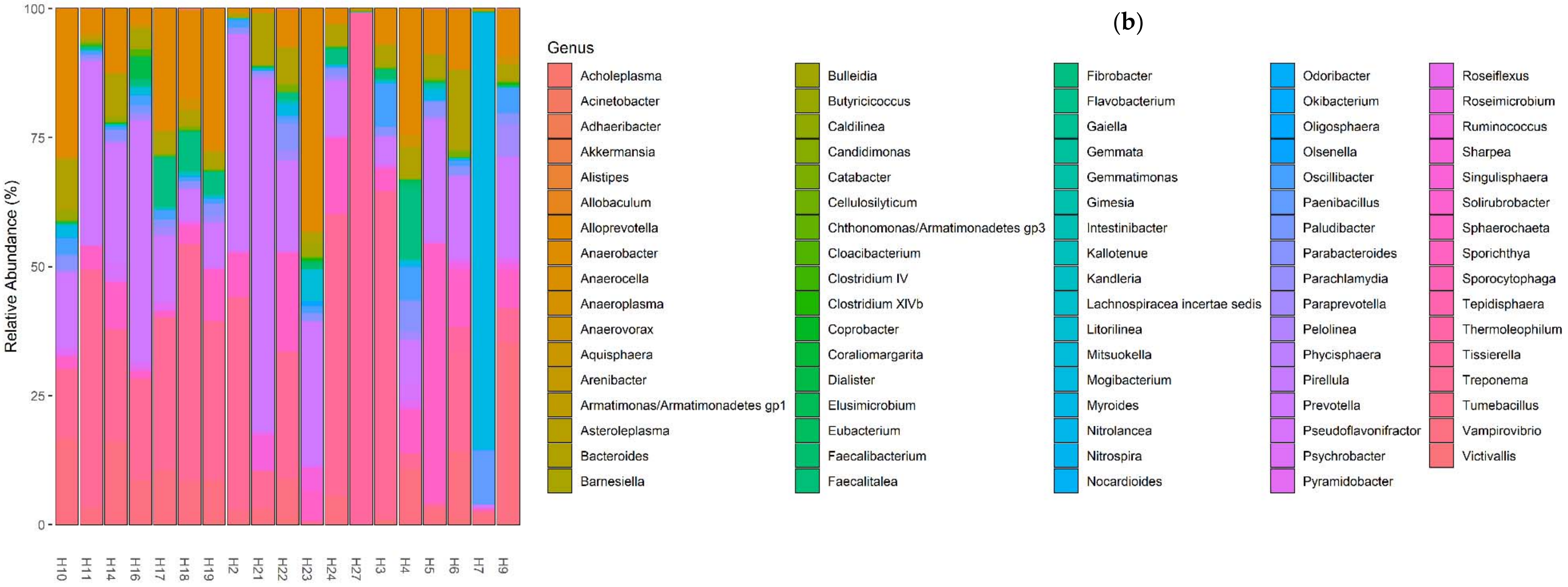

Figure 7. (**a**) Boxplot distribution of genus level relative abundance (%) observed in each 19 wild hog samples. The plot showed *Treponema*, *Prevotella*, *Alloprevotella*, *Vampirovibrio*, *Sphaerochaeta*, and *Bacteroides,* as the dominant genera in fecal microbiome of wild hogs in Texas panhandle. Data on unclassified genera were included in this plot. (**b**). Barplot distribution of genus level relative abundance (%) observed in each 19 wild hog samples. The plot showed *Treponema*, *Prevotella*, *Alloprevotella*, *Vampirovibrio*, *Sphaerochaeta*, and *Bacteroides,* as the dominant genera in fecal microbiome of wild hogs in Texas panhandle. Data on unclassified genera were included in this plot.

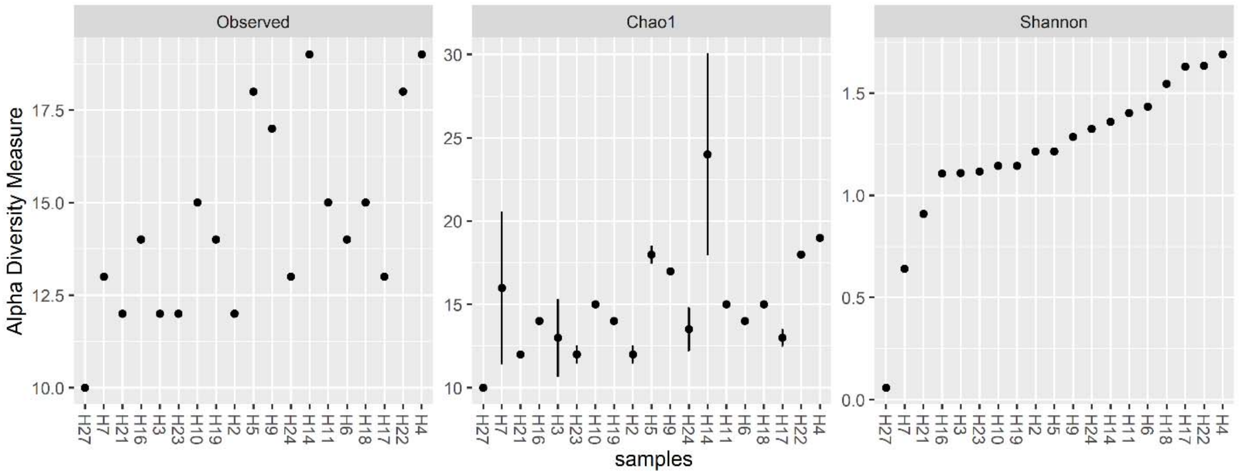

Figure S8. Alpha Diversity Index using Chao's and Shannon Indices as a measure of microbial richness and evenness within each of 19 wild hog samples. Higher alpha diversity measure represents sample(s) with greater microbial specie richness and/or evenness.

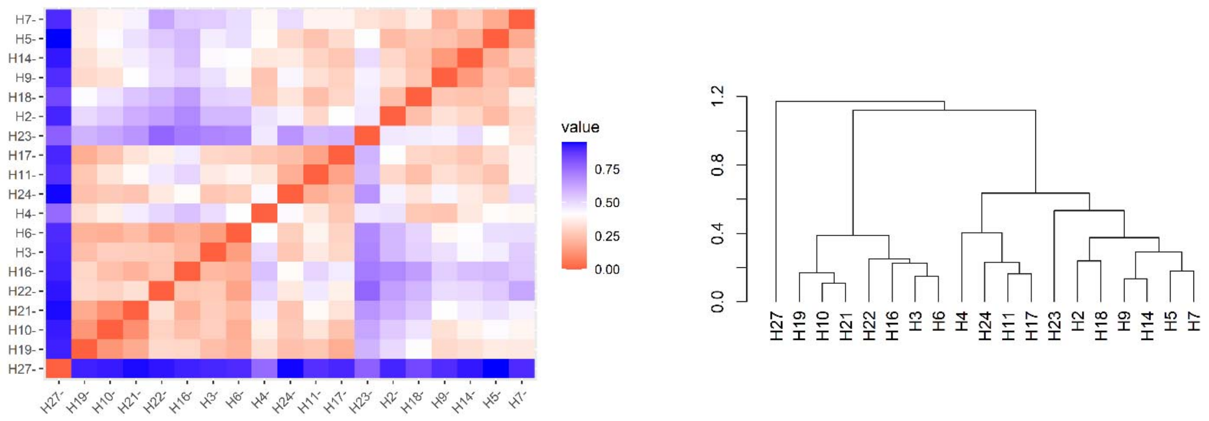

(**A**) (**B**)

Figure S9. (**A**) Bray-Curtis Dissimilarity matrix and (**B**) hierarchical clustering dendrogram as measures of beta-diversity index of wild hog fecal microbiota. **(A)** Value close to 1 showed two sample are dissimilar in microbial diversity and value close to 0 show correlation and similarity between two samples. **(B)** the higher the Ward's minimum variance value between two samples, the farther the clustering compared to another one or more samples.

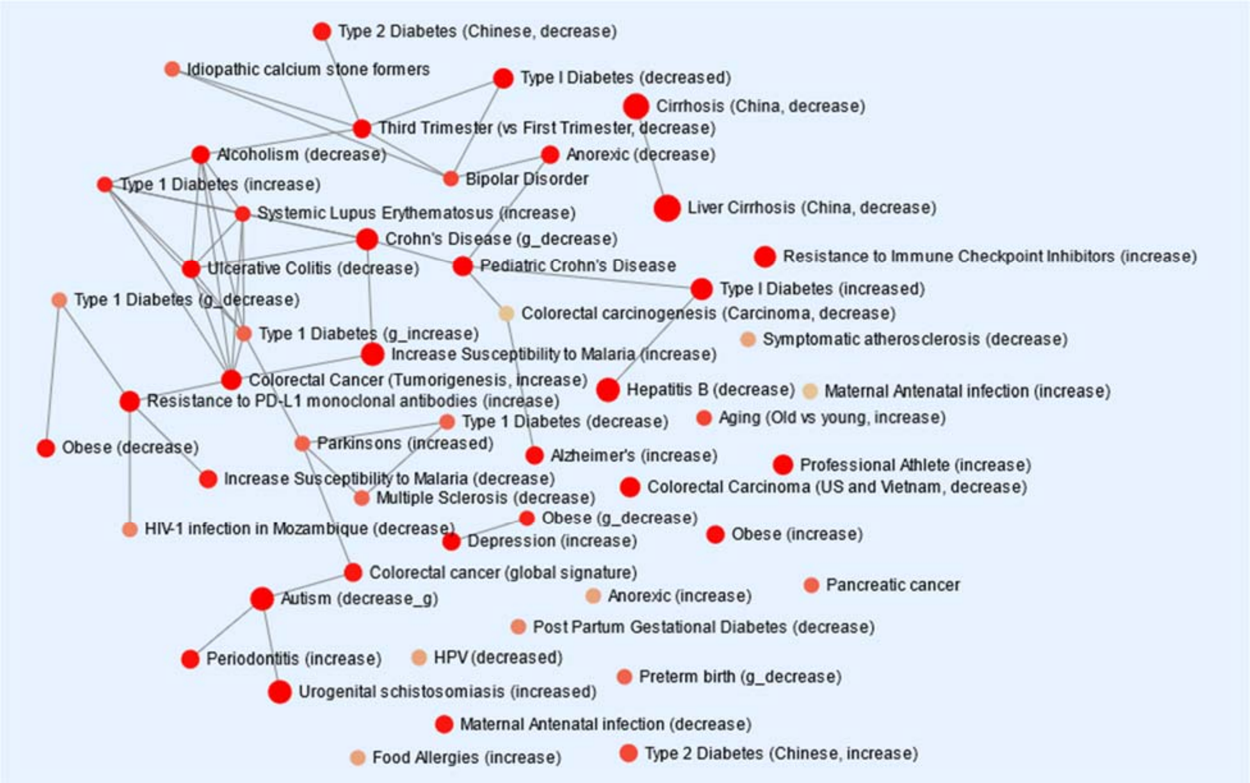

Figure S10. Predictive functional profile of wild hog microbiota in relation to certain human related diseases. Each node represents a taxon set with its color based on its p-value and its size based on the number of hits in the query. Two taxon sets are connected by an edge if the number of shared taxon hits is over 20% of the total number of their combined taxa (Chong et al., 2020).

Table S1. Statistically significant functional profile of coyote microbiota associated with certain human-related diseases.

Human-Related Diseases	Total	Expected	Hits	*p*-Value (Unadjusted)	* FDR-Adjusted *p*-Value	Associated Taxon Sets
Increase Susceptibility to Malaria (Increased risk)	8	0.163	5	1.33×10^{-7}	3.16×10^{-5}	*Barnesiella, Bacteroides, Odoribacter; Prevotella*
Hepatitis B (Decreased risk)	10	0.204	5	5.81×10^{-7}	6.91×10^{-5}	*Butyricimonas, Parabacteroides; Alistipes, Clostridium IV, Bacteroides*
Type I Diabetes (Increased risk)	13	0.265	4	8.80×10^{-5}	0.00698	*Alistipes, Bacteroides, Lactobacillus, Parabacteroides*
Autism (Decreased risk)	16	0.326	4	0.000215	0.012	*Prevotella, Treponema, Alloprevotella, Peptococcus*
Obese (Decreased risk)	7	0.142	3	0.000252	0.012	*Lactobacillus, Prevotella, Allobaculum*
Resistance to PD-L1 monoclonal antibodies (Increased risk)	8	0.163	3	0.000398	0.0133	*Bacteroides, Allobaculum, Lactobacillus*
Depression (Increased risk)	2	0.0407	2	0.000401	0.0133	*Alistipes, Oscillibacter*
Liver Cirrhosis (China, Decreased risk)	52	1.06	6	0.000446	0.0133	*Alistipes, Bacteroides, Lactobacillus, Odoribacter, Parabacteroides, Prevotella*

Increase Susceptibility to Malaria (Decreased risk)	10	0.204	3	0.00083	0.0198	*Anaeroplasma, Allobaculum, Lactobacillus*
Urogenital schistosomiasis (Increased risk)	10	0.204	3	0.00083	0.0198	*Prevotella, Alloprevotella, Peptococcus*
Colorectal Cancer (Tumorigenesis, Increased risk)	3	0.0611	2	0.00119	0.0257	*Bacteroides, Odoribacter;*
Resistance to Immune Checkpoint Inhibitors (Increased risk)	13	0.265	3	0.0019	0.0376	*Alistipes, Bacteroides, Prevotella*
Type 1 Diabetes (Decreased risk)	4	0.0814	2	0.00234	0.0429	*Prevotella, Lactobacillus*

* FDR: False discovery rate.

Table S2. Statistically significant functional profile of wild hog microbiota associated with certain human-related diseases.

**Human-Related Diseases**	**Total**	**Expected**	**Hits**	***p*-Value (Unadjusted)**	*** FDR-Adjusted *p*-Value**	**Associated Taxon Sets**
Increase Susceptibility to Malaria (Increased risk)	8	0.179	5	2.20×10^{-7}	5.23×10^{-5}	*Barnesiella, Bacteroides, Barnesiella, Odoribacter, Prevotella*
Hepatitis B (Decreased risk)	10	0.224	5	9.59×10^{-7}	6.12×10^{-5}	*Bacteroides, Parabacteroides, Alistipes, Clostridium IV, Ruminococcus*
Urogenital schistosomiasis (Increased risk)	10	0.224	5	9.59×10^{-7}	6.12×10^{-5}	*Dialister; Acinetobacter; Prevotella, Alloprevotella, Olsenella*
Crohn's Disease (Decreased risk)	5	0.112	4	1.03×10^{-6}	6.12×10^{-5}	*Bacteroides, Flavobacterium, Prevotella, Faecalibacterium,*
Cirrhosis (China, Decreased risk)	35	0.784	7	6.26×10^{-6}	0.000298	*Bacteroides, Eubacterium, Alistipes, Faecalibacterium, Parabacteroides, Odoribacter, Ruminococcus*
Liver Cirrhosis (China, Decreased risk)	52	1.16	8	9.78×10^{-6}	0.000348	*Alistipes, Bacteroides, Eubacterium, Faecalibacterium, Odoribacter, Parabacteroides, Prevotella, Ruminococcus,*
Colorectal Cancer (Tumorigenesis, Increased risk)	3	0.0672	3	1.02×10^{-5}	0.000348	*Bacteroides, Odoribacter, Akkermansia*
Autism (Decreased risk)	16	0.358	5	1.51×10^{-5}	0.000449	*Prevotella, Treponema, Alloprevotella, Olsenella, Dialister*
Type I Diabetes (Decreased risk)	4	0.0896	3	4.03×10^{-5}	0.00107	*Eubacterium, Faecalibacterium*
Resistance to Immune Checkpoint Inhibitors (Increased risk)	13	0.291	4	0.000129	0.0028	*Alistipes, Bacteroides, Prevotella*
Type I Diabetes (Increased risk)	13	0.291	4	0.000129	0.0028	*Alistipes, Bacteroides, Parabacteroides, Ruminococcus*
Pediatric Crohn's Disease (Increased risk)	7	0.157	3	0.000337	0.00669	*Bacteroides, Faecalibacterium, Ruminococcus;*

Depression (Increased risk)	2	0.0448	2	0.000486	0.00772	*Alistipes; Oscillibacter*
Ulcerative Colitis (Decreased risk)	2	0.0448	2	0.000486	0.00772	*Bacteroides, Flavobacterium*
Resistance to PD-L1 monoclonal antibodies (Increased risk)	8	0.179	3	0.000531	0.0079	*Allobaculum, Akkermansia, Bacteroides*
Alcoholism (Decreased risk)	3	0.0672	2	0.00144	0.0201	*Bacteroides; Eubacterium*
Colorectal Carcinoma (US and Vietnam, Decreased risk)	12	0.269	3	0.00196	0.0259	*Acinetobacter, Faecalibacterium, Bacteroides*

* FDR: False discovery rate.
